# Is the whole greater than the sum of its parts? De novo assembly strategies for bacterial genomes based on paired-end sequencing

**DOI:** 10.1186/s12864-015-1859-8

**Published:** 2015-08-28

**Authors:** Ting-Wen Chen, Ruei-Chi Gan, Yi-Feng Chang, Wei-Chao Liao, Timothy H. Wu, Chi-Ching Lee, Po-Jung Huang, Cheng-Yang Lee, Yi-Ywan M. Chen, Cheng-Hsun Chiu, Petrus Tang

**Affiliations:** Bioinformatics Core Laboratory, Molecular Medicine Research Center, Chang Gung University, Taoyuan, Taiwan; Institute of Biomedical Informatics, National Yang-Ming University, Taipei, Taiwan; Sequencing Technology Ltd, Taipei, Taiwan; Department of Microbiology and Immunology, Chang Gung University, Taoyuan, Taiwan; Graduate Institute of Biomedical Sciences, Chang Gung University, Taoyuan, Taiwan; Molecular Infectious Diseases Research Center, Chang Gung Memorial Hospital, Taoyuan, Taiwan

## Abstract

**Background:**

Whole genome sequence construction is becoming increasingly feasible because of advances in next generation sequencing (NGS), including increasing throughput and read length. By simply overlapping paired-end reads, we can obtain longer reads with higher accuracy, which can facilitate the assembly process. However, the influences of different library sizes and assembly methods on paired-end sequencing-based *de novo* assembly remain poorly understood.

**Results:**

We used 250 bp Illumina Miseq paired-end reads of different library sizes generated from genomic DNA from *Escherichia coli* DH1 and *Streptococcus parasanguinis* FW213 to compare the assembly results of different library sizes and assembly approaches. Our data indicate that overlapping paired-end reads can increase read accuracy but sometimes cause insertion or deletions. Regarding genome assembly, merged reads only outcompete original paired-end reads when coverage depth is low, and larger libraries tend to yield better assembly results. These results imply that distance information is the most critical factor during assembly. Our results also indicate that when depth is sufficiently high, assembly from subsets can sometimes produce better results.

**Conclusions:**

In summary, this study provides systematic evaluations of *de novo* assembly from paired end sequencing data. Among the assembly strategies, we find that overlapping paired-end reads is not always beneficial for bacteria genome assembly and should be avoided or used with caution especially for genomes containing high fraction of repetitive sequences. Because increasing numbers of projects aim at bacteria genome sequencing, our study provides valuable suggestions for the field of genomic sequence construction.

**Electronic supplementary material:**

The online version of this article (doi:10.1186/s12864-015-1859-8) contains supplementary material, which is available to authorized users.

## Background

Although many assemblers have been proposed for *de novo* assembly with NGS data, the inherent shorter length and higher error rate of short read NGS data still hinder the reconstruction of bacterial genomes. Although traditional Sanger sequencing obtained reads up to 1000 base pairs long with accuracy as high as 99.999 %, NGS generates high throughput sequences with reduced read lengths and qualities [1]. Read length is undoubtedly critical in *de novo* assembly. The most extreme example is a read as long as the entire genome that could complete construction of the entire genome. Third generation single-molecule sequencing has successfully generated reads longer than 10–15 kb or even approaching 150 kb for PacBio and MinION sequencers, respectively [2–4]. However, PacBio and MinION generate reads with sequencing errors rates as high as 15 and 35 % respectively [4, 5]. Several methods are proposed to deal with such high error rates [4–6]. Recently Illumina TruSeq Synthetic Long-Reads (previously known as Moleculo) is also proposed to provide longer reads [7]. Nevertheless, currently what we usually have reads as short as several hundreds of bps long that require sophisticated algorithms or strategies for *de novo* assembly. Many *de novo* assemblers utilizing the de Bruijn graph-based approach were developed for NGS reads, such as Velvet, SOAPdenovo, ALLPATHS, Edena, EULER-SR, and EULER-USR [8–14]. These methods construct de Bruijn graphs from short reads and obtain contigs by solving the graphs. Other studies come from a theoretical perspective and discuss whether these short reads are intrinsically sufficient for genome assembly [15, 16]. Whiteford et al. demonstrated that 90 to 97 % of the *E. coli* genome could be reconstructed with reads as short as 50 bp. However, because of repeats in the *E. coli* genome, it required read lengths of up to 475 bp to allow 99 % of the reads to uniquely map to the genome [16]. The existence of repeat sequences largely increased the complexity for resolving the whole genome sequence. The problem was only partially solved when the de Bruijn graph-based approach was proposed, and only reads longer than the repeat sequence or structural information provided by mate pair sequencing can possibly overcome this difficulty [15, 17]. In addition to reduced length, higher error rates in NGS reads also contribute to the difficulty of genome reconstruction [18, 19]. Assemblers based on the de Bruijn approach may be largely complicated by sequencing errors. Several studies have focused on providing sequencing error correction tools that can largely improve assembly results [20, 21].

Several studies have assembled whole genome sequences by combining data from both Illumina and 454 sequencing technologies that can compensate one another’s weaknesses [10, 13]. Long reads from 454 can assist short reads produced by Illumina in resolving repeat regions. As the technology rapidly advances, read length is becoming longer, and longer reads are undoubtedly helpful in the *de novo* assembly of genomes. Currently, the read length of the Illumina MiSeq has reached as long as 300 bp, although it remains shorter than the Roche 454 sequencing reads. Nevertheless, we can easily generate longer reads by overlapping Illumina paired-end reads. Several tools have already been developed for merging paired-end reads, such as the merge module in CLC, PEAR, FLASH, PANDAseq, Stitch and FastqJoin [22–27]. Paired-end read merging allows us to generate reads lengths comparable to 454. However, how these merged reads influence assembly results remains unanswered. In this study, we used real sequencing data to demonstrate the potential effects of merged reads in real world genome assembly.

To investigate the effect of merged paired-end reads in *de novo* assembly, we explored the sequence accuracy of merged reads and tried different assembly strategies with the same Illumina datasets. We generated Illumina paired-end reads with four different library sizes (300 bp, 400 bp, 500 bp and 600 bp) from the genomes of *Escherichia coli* DH1 and *Streptococcus parasanguinis* FW213. These genomes were chosen as representative bacterial genomes because they have considerably different GC contents and genome complexities, both properties known to influence sequencing quality and assembly processes. *E. coli* DH1 and *S. parasanguinis* FW213 have intermediate (50.8 %) and low (41.7 %) GC contents, respectively. *S. parasanguinis* FW213 has a higher proportion of short tandem repeats and long repeats in its genome compared with *E. coli* DH1. We explored the accuracy of these reads and the *de novo* assembly results from these reads. Although MiSeq has been shown to provide the lowest error rate compared with the 454 GS Junior and Ion Torrent PGM, which both produce homopolymer-associated indel (insertion/deletion) error [28], we still observed certain levels of sequencing errors, even after reads were trimmed. We also observed that these errors could be significantly reduced by simply merging paired-end reads. However, the merge step sometimes also largely increased indel errors, particularly for reads from libraries with few overlapping regions or from genomes with many tandem repeats. Given that the merged reads had higher sequence qualities and longer lengths, we further performed reconstruction of the genome sequences using different *de novo* assembly strategies with either paired-end reads or merged reads for different library sizes. In addition to assembly strategies, information on how many reads should be used will be helpful in experimental design. Several studies already provide suggestions on the optimum depth for genome assembly [29, 30]. By examining the assembly results generated from real data, we discuss how paired-end reads should be used to generate the best assembly results. Our study provides invaluable and useful suggestions for hundreds of ongoing bacterial genome reconstruction projects.

## Methods

### Sequence read preparation

Total cellular DNA from *E. coli* DH1 and *S. parasanguinis* FW213 were prepared by standard protocols [31]. Four paired-end libraries containing different sizes of DNA fragments (300 bp, 400 bp, 500 bp and 600 bp) were prepared by Ovation Ultralow Library Systems (NuGEN) for both *E. coli* DH1 and S. *parasanguinis* FW213. The libraries were sequenced by an Illumina MiSeq sequencer. A total of eight different datasets of paired-end 2 X 250 bp reads were generated (SRP060735). For sequence quality estimation, calculate_stats from seq_crumbs was used to estimate the read qualities for each dataset [32]. For further *de novo* assembly analysis, reads were trimmed to fit the quality threshold of Phred quality scores larger than Q20 and lengths larger than 50 bp.

### Mapping reads to reference genomes

The reference genome sequences of *E. coli* DH1 (NC_017625) and *S. parasanguinis* FW213 (NC_017905) were downloaded from NCBI [33]. We used seqtk [34] to randomly select 40 % of all original raw reads 5 times. These subsets of raw reads were merged with or without trimming. The resulting dataset of reads (raw reads, trimmed reads and merged reads) were all mapped back to their corresponding reference genomes with BWA-MEM using default parameters [35]. Mismatch and mapping rates were calculated from the output mapped bam files using sam-stats from ea-utils [24].

### Overlapping paired reads

To investigate the effect of overlapping reads, the “merge overlapping pairs” module in the CLC Genomics workbench was used to overlap the paired-end reads [26]. For reads that could be overlapped together (referred to as “Group M” henceforth), their overlapping reads and original paired-end reads were both generated as separate files using several in-house scripts.

### *de novo* assembly

The CLC Genomics Workbench [26] was used for *de novo* assembly with default parameters. There were five different assembly strategies. The original raw reads were assembled as paired-end reads (Group A [PE]) or as single-end reads (Group A [SE]). Reads that overlapped were either assembled as paired-end reads (Group M [PE]) or as single-end reads (Group M [SE]) after overlapping. All of these merged single-end reads and the rest of the non-overlapped paired-end reads were also assembled together (Group A [PE + SE]). For each assembly strategy, an in-house, multithreaded Perl script was used to randomly generate different numbers of reads from the total number of reads. These reads were randomly sampled from the raw data, and 9260/4340 paired reads were treated as coverage depths of 1X for *E. coli* DH1 and *S. parasanguinis* FW213 which have genome sizes of 4.63 Mb and 2.17 Mb, respectively. We randomly selected three independent datasets from each depth to estimate variance.

### Repeat region analysis

The Tandem Repeats Finder was used to identify tandem repeats in both the *E. coli* DH1and *S. parasanguinis* FW213 [36] genomes. Only tandem repeats with matches larger than 80 % were included and analyzed. For short tandem repeat analysis, tandem repeats with sizes smaller than 100 bp were included. These tandem repeat period sizes were multiplied by their corresponding copy numbers and then summed to estimate the percent of the genome that was located within tandem repeats.

### Estimating assembly quality

All contigs generated from *de novo* assembly were further evaluated by QUAST [37]. From the QUAST report, several important and representative statistical values, such as N50, genome fraction (%), number of misassemblies, number of genes covered, and number of contigs and number of indels per 100 kb were used to evaluate the assembly qualities.

## Results and discussion

### Merging paired-end reads improves their accuracy

To investigate the effect of overlapping paired-end reads on the accuracy of merged reads, we generated eight different datasets from the *E. coli* DH1 and *S. parasanguinis* FW213 genomes. We produced 2 X 250 paired-end reads from each genome. We designed four different library sizes: 300 bp, 400 bp, 500 bp and 600 bp for each genome, and all reads were generated by the MiSeq platform. All eight datasets contained more than 1 million reads and high sequence quality, with average Phred scores greater than 30 (Table 1). The CLC workbench was then used to merge overlapped paired reads. This merging step looked for overlapping regions from the ends of paired reads and merged these overlapped regions. If any conflicts in the overlapped region existed, the read with the higher quality score was used. We defined two read datasets from this merging step: Group A that included all reads and Group M that included only a subset of reads that could be merged. As expected, most of the reads from the library size of 300 could be merged together, and the percentage of read pairs that could be merged decreased as the library size increased (Table 1).

**Table 1:** Summary of paired–end reads used in this study

Reference genome	Library size	Read number	Average quality^a^	Q20^b^	Q30^b^	Group M^c^
*E. coli* DH1	300 bp	1,894,068	34.31	90.15 %	85.27 %	71.59 %
*E. coli* DH1	400 bp	2,221,312	33.91	88.94 %	83.61 %	69.21 %
*E. coli* DH1	500 bp	2,093,116	33.50	87.63 %	81.87 %	25.12 %
*E. coli* DH1	600 bp	1,548,836	32.79	85.29 %	78.78 %	0.85 %
*S. Parasanguinis* FW213	300 bp	2,162,006	35.84	93.47 %	90.77 %	82.05 %
*S. Parasanguinis* FW213	400 bp	1,968,178	35.60	92.67 %	89.76 %	81.04 %
*S. Parasanguinis* FW213	500 bp	1,886,084	35.21	91.55 %	88.26 %	19.03 %
*S. Parasanguinis* FW213	600 bp	1,745,876	34.61	89.58 %	85.67 %	6.82 %

^a^Average Phred score

^b^The percentage of bases in the reads with a Phred score equal or larger than 20 or 30

^c^Percentage of paired reads that can be merged

**Fig. 1:**
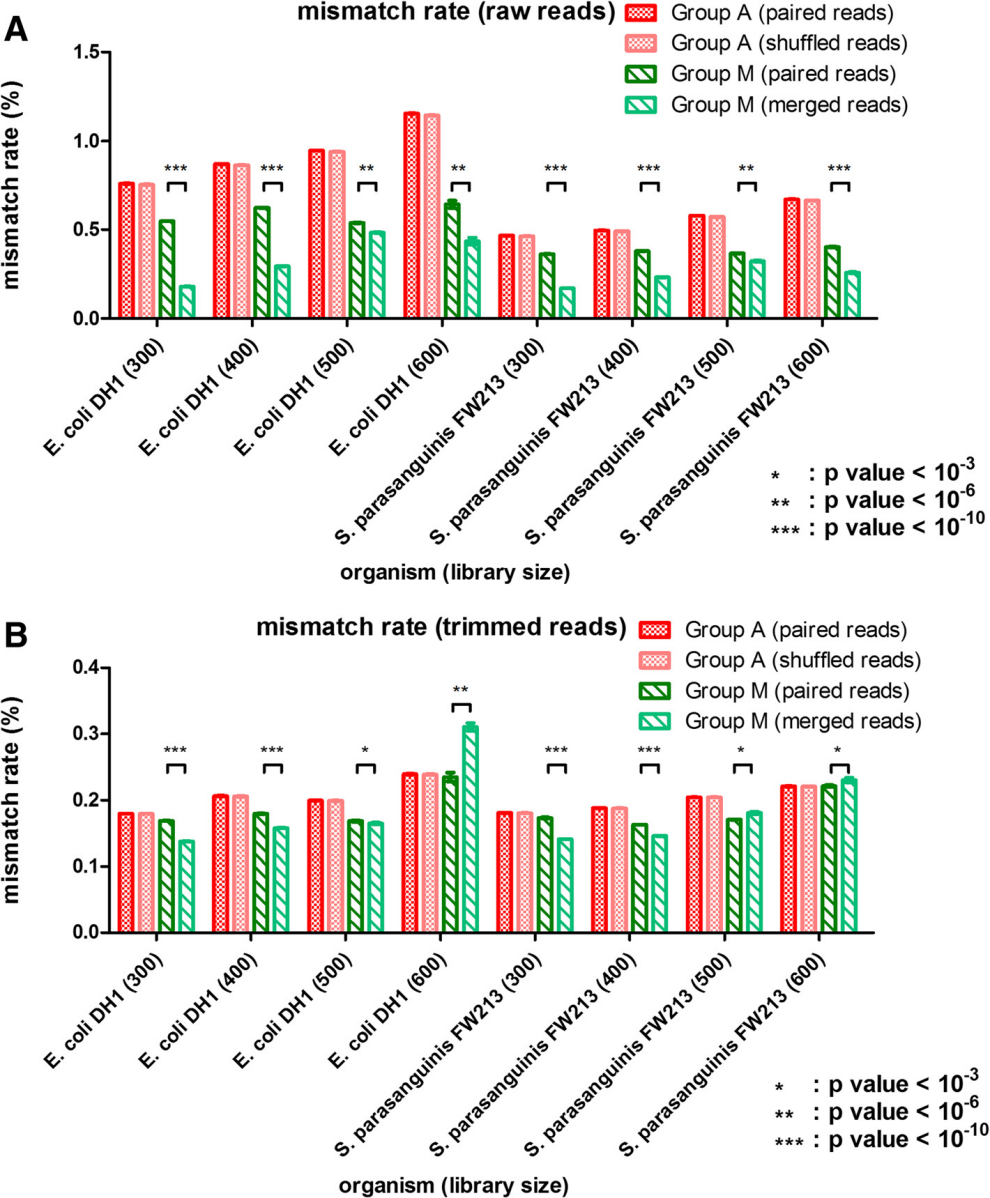
Mismatch rates detected from mapping reads to reference genome. Reads from Group A (all reads) were mapped to reference genomes as paired-end reads or single end reads (R1 and R2 shuffled together). Reads from Group M (reads can be merged together) were mapped as paired-end reads or merged single reads. Mismatch rates for Group A and Group M are explored. For raw reads (**a**), the mismatch rates of Group A were similar for both paired-end mapping and shuffled reads mapping. The rate decreased significantly for Group M (paired-end) and decreased even more for Group M (merged reads). For trimmed reads (**b**), there was much less decrease or even increase of mismatch for large libraries (500 bp and 600 bp). The difference of mismatch rates between Group M (paired reads) and Group M (merged reads) was tested by paired t tests

**Fig. 2:**
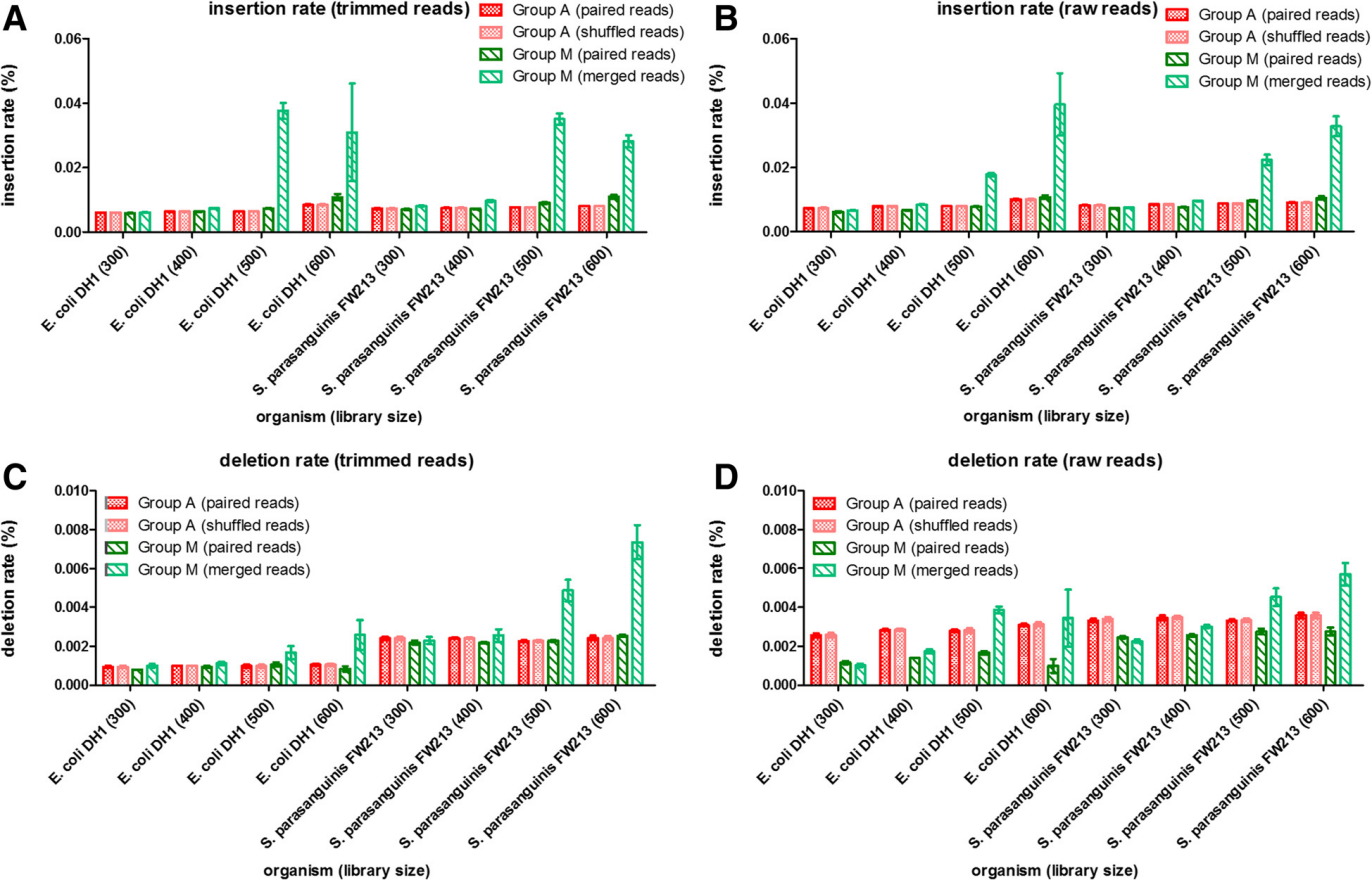
Insertion and deletion rates detected from mapping reads to reference genome. After reads mapped to reference genome, insertion and deletion rates were estimated. Insertion rates for trimmed and raw reads are shown in (**a** and **b**), respectively, and deletion rates are shown in (**c** and **d**), respectively. Trimmed reads from Group A always have slightly lower insertion and deletion rates than raw reads. However, the merged reads have significantly higher insertion and deletion rates particularly for these from large library (500 bp or 600 bp). For each group, we subsampled the reads 5 times and plot their standard deviation

The influence of sequence accuracy after the merging step was the first issue we investigated. Because base quality usually drops towards the end of the read, some sequencing errors can be corrected during the merging step. To survey the extent and effect of correcting sequencing errors in this manner, we mapped raw paired reads and merged reads from Group M to the reference genomes. We observed that the mismatch rate for Group M was significantly lower than that for Group A (Fig. 1). This result demonstrates that only raw reads with higher accuracy could be merged. Although the merged reads did not extensively increase the mapping rate, they did significantly decrease the mismatch rate. Although the merging step only involved partial read regions, this step corrected 10.2 to 67.3 % of the mismatches in raw reads (Additional file 1: Table S1). The accuracy improvement was most significant in the smallest library that had the longest overlapping regions on average (Fig. 1a). We further examined whether the same sequence accuracy improvement could be achieved by sequence trimming, which is one of the most commonly used strategies for eliminating low quality bases. Raw reads were trimmed for quality (Phred score larger than Q20) and length (longer than 50 bp) and then mapped back to the reference genomes with and without the merging step. We still observed a significant decrease in mismatch rate for trimmed paired-end reads from library sizes of 300 bp and 400 bp but an opposite trend for reads from larger libraries (Fig. 1b). Similar decrease and increase pattern in mismatch rates were found when reads were merged by FLASH, PANDAseq or PEAR (Additional file 1: Table S2). This increase in mismatch rate may be random because fewer reads belonged to Group M for large library size. In our example, the number of reads from the 600 bp library of Group M was approximately 1/100 (*E. coli* DH1) and 1/10 (*S. parasanguinis* FW213) the number of reads from other library sizes. This small size in reads number make this group more susceptible to sampling fluctuation. However, the merge step may introduce incorrect sequences by erroneously merging non-overlapping region. Because these mistaken merges were most likely to occur in regions with repeats, we further explored the insertion and deletion rates from previous mapping results (Fig. 2). We observed that reads from Group A had similar insertion and deletion rates whether mapped as paired-end or single-end reads. However, both the insertion and deletion rates significantly increased for merged reads from Group M with library sizes of 500 bp and 600 bp. Increase in both insertion and deletion rates were also found when reads were merged by FLASH, PANDAseq or PEAR (Additional file 1: Table S3 and Table S4). This result implies that when the overlapped regions are short or non-existent, the merging step may cause a significant number of indel errors, explaining the increase of the mismatch rate observed for the merged reads from large library sizes.

### Location information from paired-end reads largely promotes *de novo* assembly

**Fig. 3:**
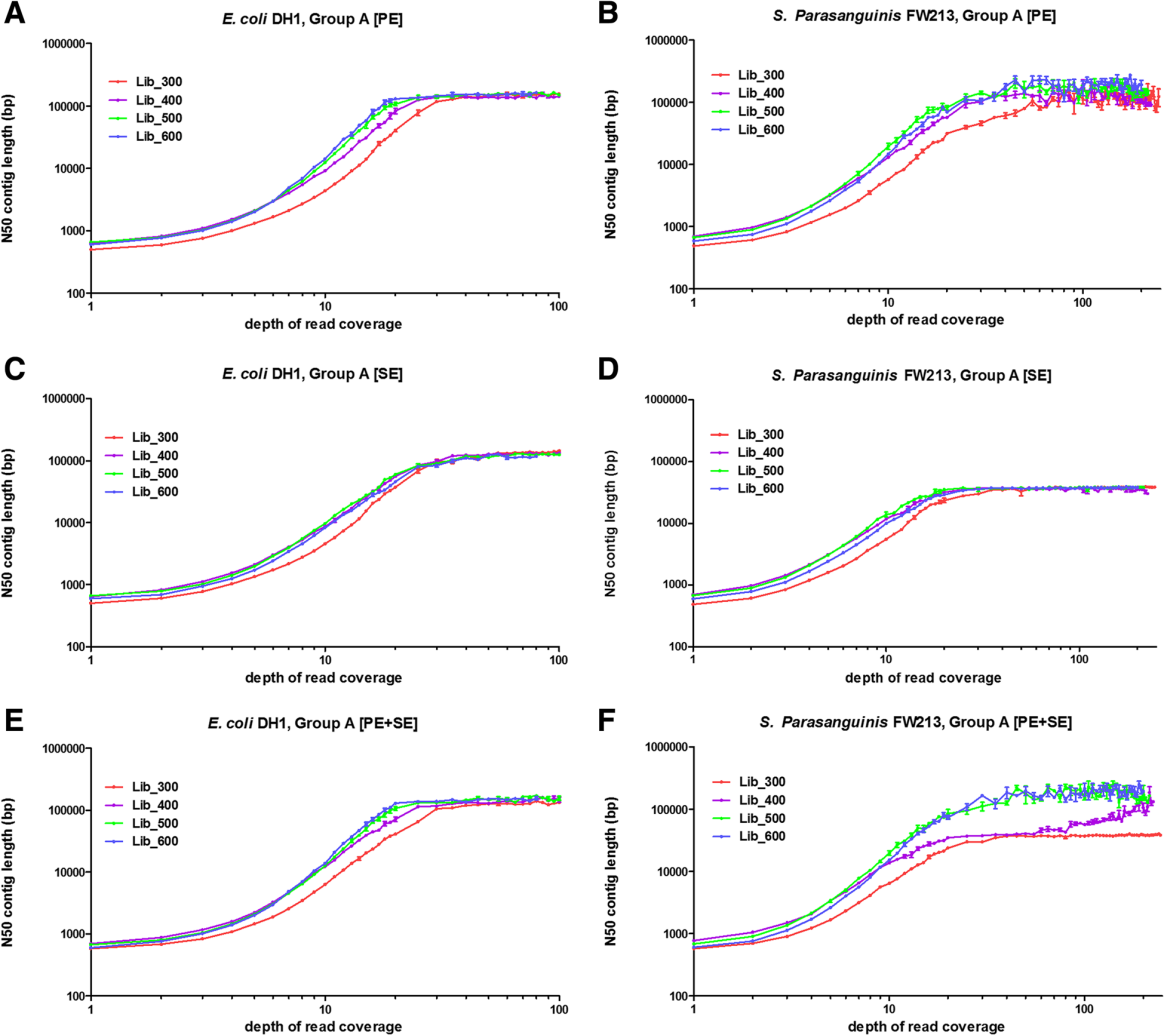
N50 of assembly results for different library sizes. N50 values for four different library sizes (300 bp, 400 bp, 500 bp and 600 bp) for *E. coli* DH1 and *S. Parasanguinis* FW213 are shown together with their standard error of the mean for different depths of reads. **a** and **b** are N50 from Group A [PE] and **c** and **d** are N50 from Group A [SE] which are results from all reads assembled as paired-end reads and single end reads, respectively. **e** and **f** illustrate the results from Group A [PE + SE] which assembled all the non-overlapped paired-end reads and merged reads altogether. From A and B, we observed that larger library size tended to produce larger N50 values when assembled as paired-end sequence but not when assembled as single-end sequence (**c** and **d**). These increases in values were attributed to the utilization of pair-end information

Based on the improved accuracy observed with the overlapped paired-end reads, we were interested in investigating how the improvement influences the process of *de novo* assembly. For all library sizes, we assembled paired raw reads as paired-end reads (Group A [PE]) or as single-end reads (Group A [SE]). We also assembled the merged reads together with the non-overlapping reads (Group A [PE + SE]). To compare the assembly results for different library sizes, we used N50 (N50 is the length for which the collection of all contigs of that length or longer contains at least half of the sum of the lengths of all contigs) to represent the integrity of assembly result [38]. As shown in Fig. 3, as read number increased, N50 increased and then plateaued. Within all assembly strategies, Group A [PE] always yielded the highest N50 for all library sizes. Group A [PE + SE] had similar performance as Group A [PE] in *E. coli* DH1 but did not generate a higher N50 value compared to Group A [PE]. Nevertheless, Group A [PE + SE] had significant drops of N50 were observed for library sizes of 300 bp and 400 bp in *S. parasanguinis* FW213 (Fig. 3f). This drop also existed in large libraries (500 bp or 600 bp) that had much lower ratios of overlapping reads.

Larger library size typically yielded a higher N50 value given the same read number and assembly strategy (Fig. 3a and b). We also examined the number of misassemblies for assembled contigs from different library sizes. The rate of misassemblies is not higher for these assembled results with higher N50 (Additional file 2: Figure S1). This result suggest the higher N50 values really represent better assembly results. Because quality scores for different library sizes were similar (Additional file 1: Table S5), the advantage observed in libraries with longer sizes likely resulted from the paired-end information but not read quality. To investigate whether the higher N50 values for larger library sizes can be attributed to the paired-end information alone, we further investigated the results from Group A [SE] (Fig. 3c and d), in which the superiority of larger libraries disappeared. We noticed that the smallest library (300 bp) still had a slightly lower N50 value because most read pairs from this library have almost overlapped with each other completely. However, Group A [SE] had much worse d*e novo* assembly results compared with Group A [PE] in all datasets, again indicating the importance of location information in the *de novo* assembly process. Similar patterns were found when we used different assemblers (IDBA-UD and SPAdes) as shown in Additional file 3: Figure S2 [39, 40].

### Merged reads improve *de novo* assembly only when read depth is low

**Fig. 4:**
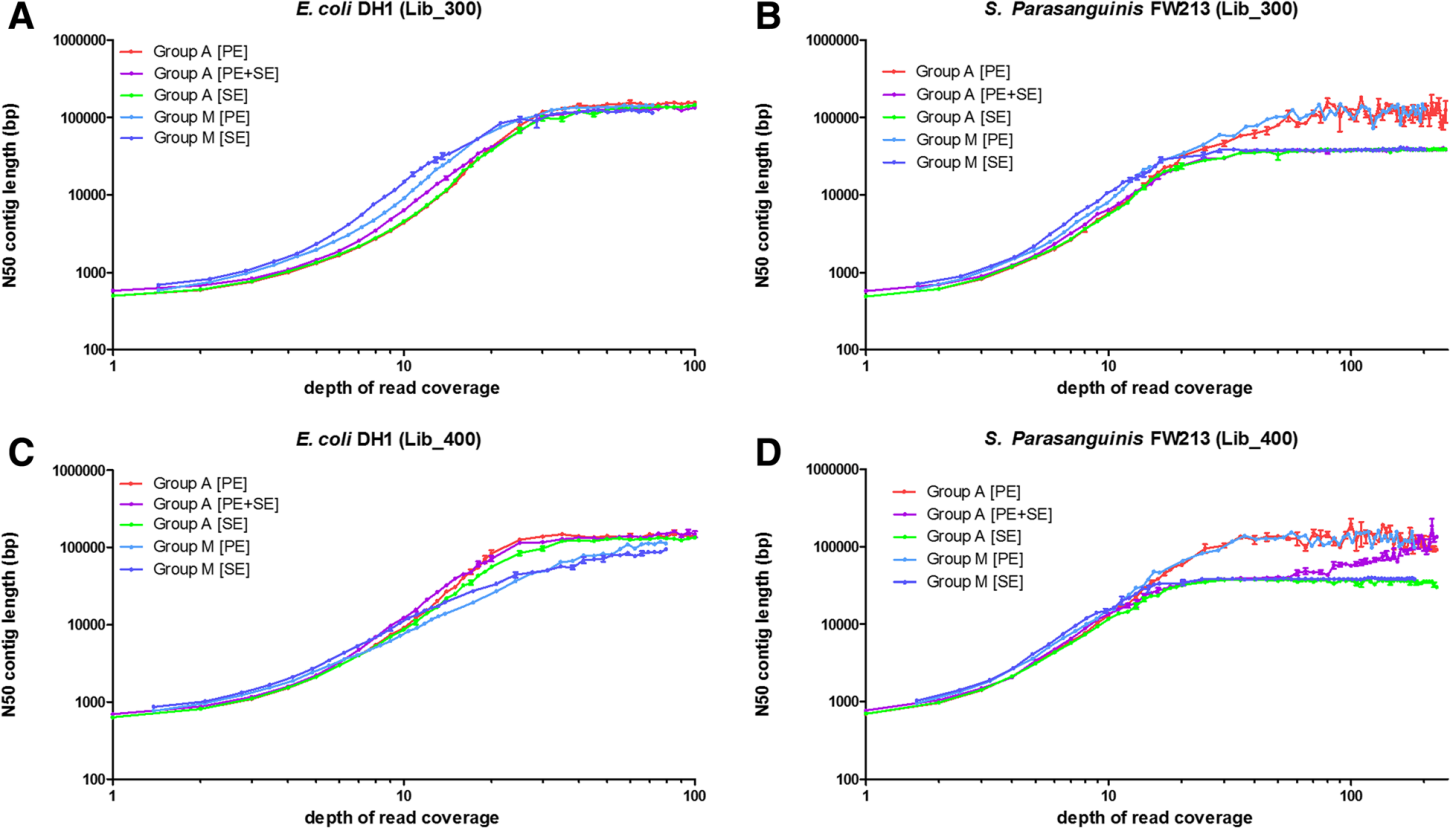
N50 of assembly results for different assembly strategies. N50 values for the *de novo* assembly results for *E. coli* DH1 and *S. Parasanguinis* FW213 are shown together with their standard errors of the mean. Group A [PE] and Group A [SE] represent all reads assembled as paired-end reads and single end reads, respectively. Group A [PE + SE] represents all the non-overlapped paired-end reads assembled together with merged reads. Group M [PE] and Group M [SE] represent Group M reads assembled as paired-end reads and single end reads, respectively. Although Group A [PE] always had a higher or similar N50 compared with Group A [SE], Group M [SE] yielded a higher N50 than Group M [PE] when the depth of read coverage was low

To further examine the effect of merged reads in the *de novo* assembly process, we focused on the subset of reads that could be merged (Group M). We assembled Group M as paired-end (Group M [PE]) or merged single end reads (Group M [SE]). The two large libraries (500 bp and 600 bp) were skipped in this analysis because few overlapping reads were present in these two groups. We observed that Group M [PE] and Group M [SE] had similar performances on the *E. coli* DH1 genome in terms of N50 and number of misassemblies (Fig. 4a and c and Additional file 4: Figure S3). However, the N50 from Group M [PE] was higher than the N50 from Group M [SE] in *S. parasanguinis* FW213 (Fig. 4b and d) when the depth of read coverage was higher than 20X. We suggest that this difference may be because of specific properties of the genome—in particular, the presence of tandem repeats that can increase the possibility of erroneously merged reads and hinder assembly. Because short tandem repeats may become sources of erroneous merges, we focused on tandem repeats shorter than 100 bps. We observed that 0.68 % of the *S. parasanguinis* FW213 genome was covered by tandem repeats compared with only 0.13 % of the *E. coli* DH1 genome. Two of the tandem repeats, 72 bp and 36 bp, on the *S. parasanguinis* FW213 genome had copy numbers as high as 81 and 165, respectively. Although such high copy numbers are infrequent, S*. parasanguinis* FW213 has 5.4 times more tandem repeats than *E. coli* DH1. This higher rate of repeats also explains the higher insertion and deletion rates for *S. parasanguinis* FW213 in Fig. 2. The merging step may introduce errors into the middle of reads that will largely complicate the de Bruijn graph used in assembly and result in incorrect assembly results in *S. parasanguinis* FW213.

When the depth of read coverage was small, Group M outcompeted Group A (Fig. 4). This difference may result from the slightly higher overall quality of reads from Group M compared with reads from Group A (Additional file 1: Table S6). Given that Group M only included reads that had overlapped regions, these reads consequently provided less location information. This result suggests that when the depth of read coverage is low, read quality is a critical factor in *de novo* assembly (Fig. 4). However, as the depth of read coverage increased, paired-end information could compensate and eventually provide greater benefits than higher accuracy. Similar results were observed when these reads are assembly by IDBA-UD or SPAdes (Additional file 5: Figure S4). For Group M, merged single end reads yielded an even higher N50 than the original paired-end reads (blue and light blue lines in Fig. 4). This result indicates that longer reads with higher accuracy improve the assembly results. Nevertheless, this improvement again disappears when the depth of read coverage increases. We observed that Group M [SE] barely overcame Group M [PE] in *S. parasanguinis* FW213. We believe that this result is because of the differences in the genome complexities of *E. coli* DH1 and *S. parasanguinis* FW213. Although *S. parasanguinis* FW213 has a smaller genome, it contains 7 tandem repeats larger than 400 bp, and these repeats have copy numbers range from 2.2 to 6.1 (Additional file 1: Table S7). Under the same criteria, *E. coli* DH1 only has a repeat of 535 bp with a copy number of 2.9.

### Merged reads increase indel frequency in *de novo* assembly when depth of read coverage is low

**Fig. 5:**
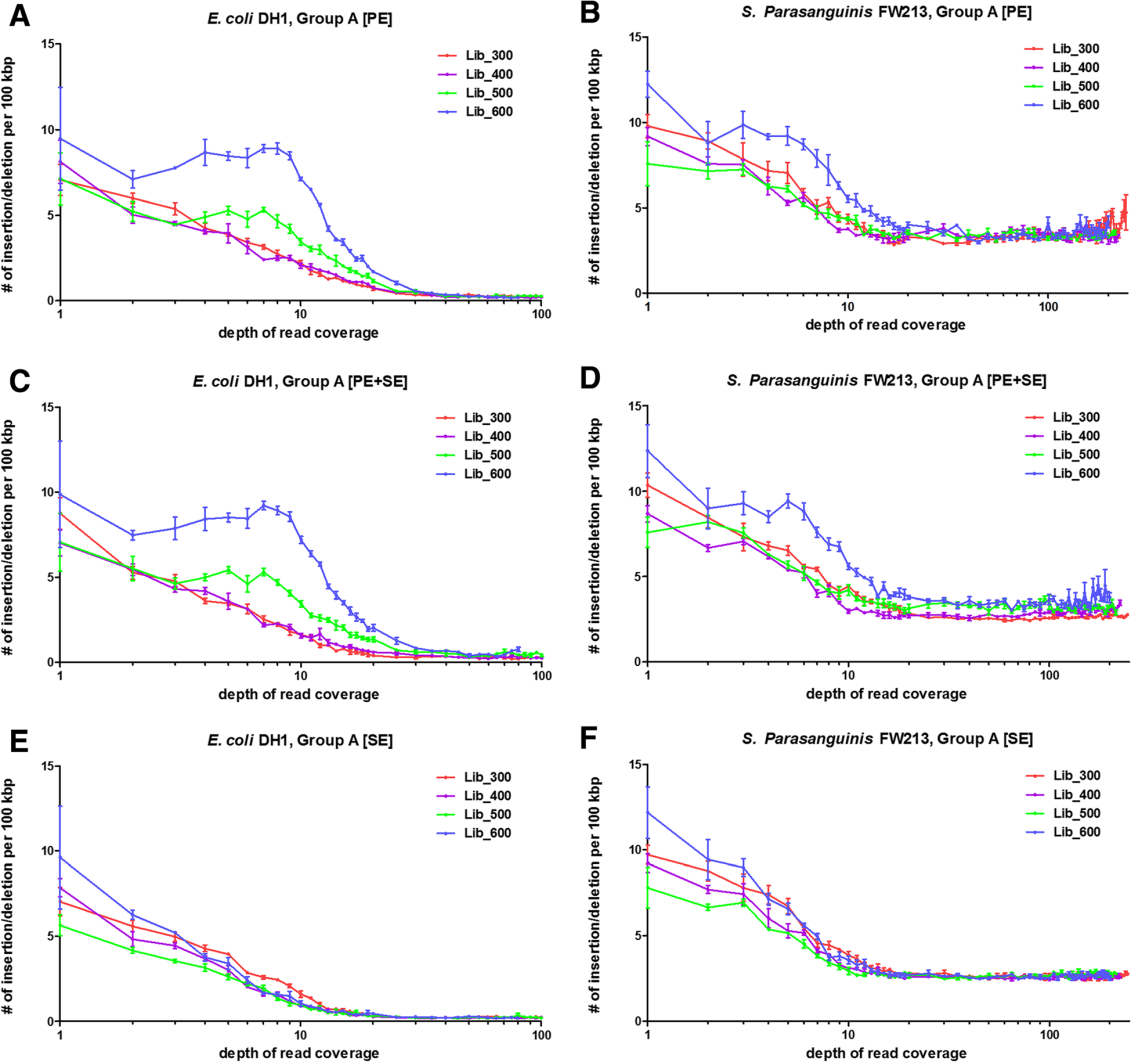
Number of indels per 100 kb for different assembly strategies. Numbers of insertions/deletions (indels) for Group A are plotted with different depths of reads. Four different library sizes (300 bp, 400 bp, 500 bp and 600 bp) for both *E. coli* DH1 and *S. Parasanguinis* FW213 are shown in different colors. For almost all assemblies, the numbers of indels tended to decrease as increasing number of reads were used for *de novo* assembly to a certain point. The minimum limit for *S. Parasanguinis* FW213 was much higher than that for *E. coli* DH1

Because the merging step may mistakenly introduced indels into reads, we further explored the indel rates for these assembly results. By comparing assembled contigs with reference genome we can obtain the number of indel in our assembled contigs for different assembly strategies of Group A (Fig. 5). We found that the number of indels decreases as more reads used in the assembly. This is reasonable because when depth of read coverage is low, errors in few reads may result in errors in de Bruijn graph that can be corrected when more reads are used to construct the graph. We also observed higher indel rates for large library (600 bp) when comparing between different library sizes. Although the indel rates tend to decrease as the depth of read coverage increases, there are exceptions in both PE and PE + SE groups when depths of reads are low (Fig. 5). This phenomenon suggests that even if the reads were merged, the similarity in the end of read pair causes indels in the final assembly result when depths of read coverage are not high enough. It is worth mentioning that the indels rates are slightly lower in PE group comparing to PE + SE group. This result suggests the error indels in assembled contigs are more likely to be created when paired information are used from barely overlapped paired-end reads and the merging step may augment the error rate. Nevertheless most of these indel errors can be eliminated when the read number is large enough. Even the depth of read coverage for *S. parasanguinis* FW213 reached more than 200X, twice the maximum depth of *E. coli* DH1. We observed that the number of indels in *S. parasanguinis* FW213 was higher than that in *E. coli* DH1. The higher indel rate is again consistent with the idea that the elimination of indels is more difficult for genomes containing repeats. This result demonstrates that these repeats can only be resolved by reads longer than the repeats instead of increasing depth of read coverage.

### Gene and genome coverage and sequencing depth

**Fig. 6:**
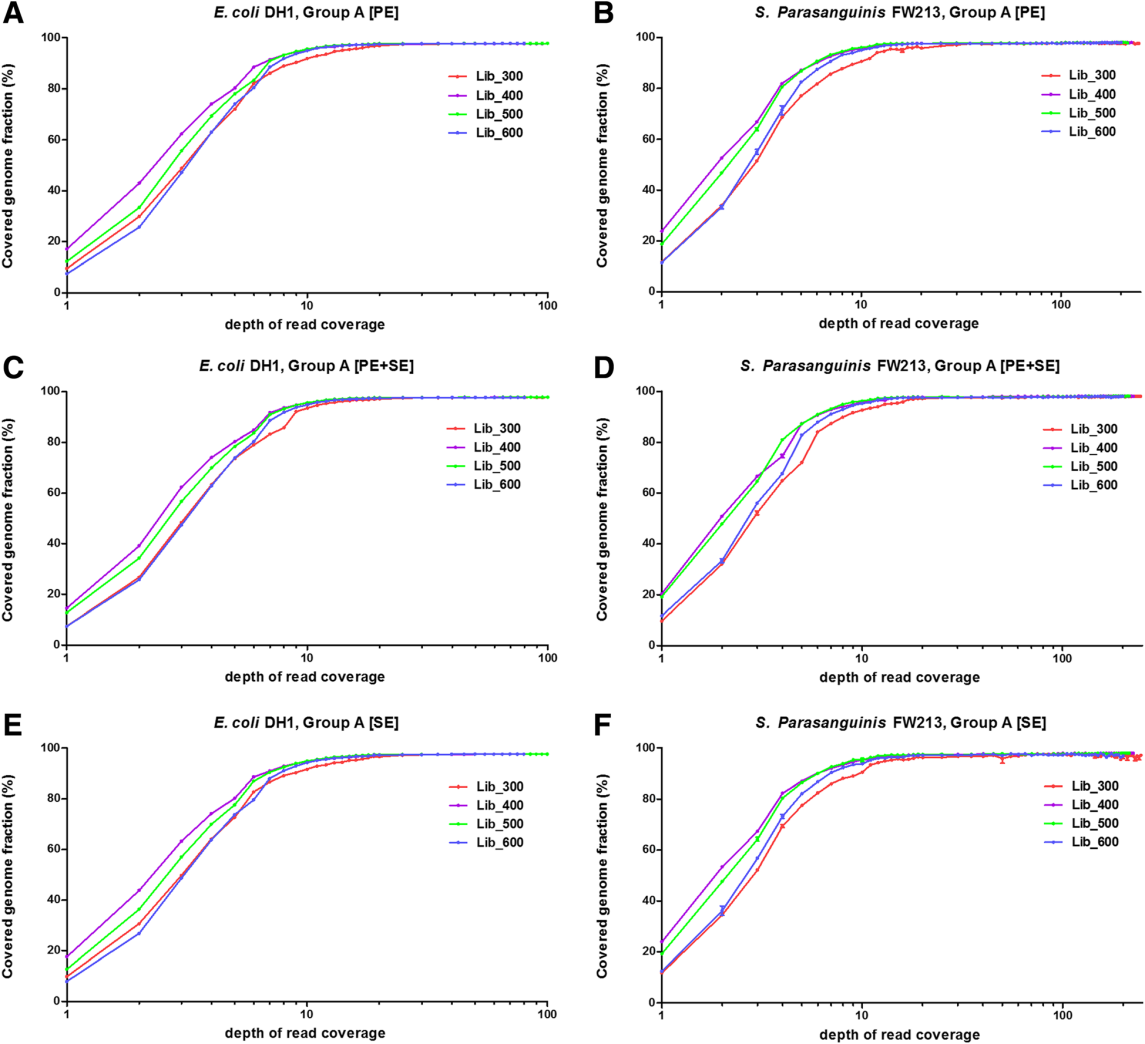
Percentage of genome covered by contigs. For different assembly strategies, the percentages of the genomes covered by the resulting contigs are plotted against depth of read coverage for *E. coli* DH1 and *S. Parasanguinis* FW213. Four different library sizes (300 bp, 400 bp, 500 bp and 600 bp) are shown in different colors. There was no large difference for different assembly strategies. The median library sizes covered higher genome fraction when the depth of read coverage was low

By examining the *de novo* assembly results from real sequencing data, we further explored how many reads are required for *de novo* assembly analysis. Although the optimal sequencing depth may vary for different read length and organisms, our data can provide hints for analysis of genomes with similar characteristics. We explored how much of the genome is covered by assembled contigs. The covered genome fraction increases as sequencing depth increasing and finally reaches plateau. The genome coverage is different for different library sizes and the median library sizes (400 bp and 500 bp) tend to have relatively higher genome coverage fraction while depth of read coverage is low. However, as sequencing coverage depth increasing all libraries can reach to similar plateau value. We found that 97 % of the genome can be covered by coverage depths approximately 12X-19X and 19X-50X for the larger libraries (400 bp, 500 bp and 600 bp) and smallest library (300 bp), respectively (Fig. 6). We also found that the plateau value is approximately 96.2 ~ 98.2 %, which again supports previous results that part of the genome can never be resolved until reads larger than the long repeat regions are included in *de novo* assembly analysis. We also examined the covered genome fraction across different assembly strategies (Additional file 6: Figure S5). We found the group M has slightly higher coverage while the depth of read coverage is low but as depth of read coverage increasing finally all assembly strategies all reach plateau with similar covered genome fraction. We further explored how many of the annotated genes were fully covered by these assembled contigs. The pattern of gene coverage was similar to genome coverage only with relatively lower coverage that was obvious because only fully covered genes considered. The plateau value for gene coverage was 92.9 ~ 98.2 %.

Another suggestion for choosing read coverage depths is to generate a higher number of reads than actually necessary and then obtain a subsample from original reads. We noticed that there are fluctuations for N50 values in all assembly strategies particularly for large coverage depths (Figs. 3 and 4,). Although all of these read numbers are the same and all of the reads were already selected with their read quality and read length, different combinations of these qualified reads resulted in different assembly results. Regardless of the cause of the difference, our result suggests that when the number of reads is large enough, obtaining subsets from all of the available reads and performing multiple *do novo* assemblies might be beneficial. From the same dataset, certain combinations of reads may provide better genome reconstruction results. The reason behind the differences in assembly results may be the sequence content of the reads, the real covered regions in the genome or other factors. Thus, further study is warranted to generate selection criteria for genome assembly studies.

## Conclusions

As more and more bacteria genome reconstruction projects are conceived, it is increasingly more important to have guidelines and suggestions for designing the analyses. In this study, we provide detailed paired-end read analyses of real sequencing data from two bacteria and generate some useful suggestions for future bacterial genome analyses. By using four different real datasets from two representative genomes, we investigated the effect of merging overlapping paired-end reads. We observed that the merging step could create longer reads with higher accuracy. We further demonstrated that the improvement in read accuracy could improve assembly when reads were fewer. However, as the read number increased, paired-end information rather than sequence length or quality is the key factor dominating the outcome of *de novo* assembly. In addition, we also observed that the merging step may unexpectedly create indels within reads that further create indels in assembled contigs. Although these indels in contigs can be eliminated when the depth of read coverage increases, the elimination is less effective for genomes with many repeats. In summary, our results suggest that merging paired-end data analysis is not always beneficial and better avoided when the genome under study contains many repeat sequences. These results shed new light on genomic sequence construction.

## Additional files

Additional file 1: Table S1-S7.
**Table S1** – Summary of mapping reads to reference genome. **Table S2** – Mismatch rates detected from reads mapped to reference genome before and after merge by different merge programs. **Table S3** – Insertion rates detected from reads mapped to reference genome before and after merge by different merge programs. **Table S4** – Deletions rates detected from reads mapped to reference genome before and after merge by different merge programs. **Table S5** – Q30* values for Group A from different libraries. **Table S6** – Q30* values for Group M from different libraries. **Table S7** – Tandem repeats in *S. Parasanguinis* FW213. (DOCX 36 kb) Additional file 2: Figure S1. Number of misassemblies for assembly results from different library sizes. Number of misassemblies for four different library sizes (300 bp, 400 bp, 500 bp and 600 bp) for *E. coli* DH1 and *S. Parasanguinis* FW213 are plotted against different depth of read coverage. A and B are from Group A [PE] which represents assembled results from all reads assembled as paired-end reads. C and D are from Group A [PE + SE] which represents assembled results from merged reads and all the non-overlapped paired end reads. E and F are from Group A [SE] which represents assembled results from all reads assembled as single-end reads. The number of misassemblies decreases as the depth of read coverage increases. Even though there are some fluctuations when depth of read coverage is low, the number of misassemblies reaches a steady number for all library sizes when depth of read coverage is high. (TIFF 882 kb) Additional file 3: Figure S2. N50 for assembly results from different library sizes assembled by IDBA-UD and SPAdes. N50 values for four different library sizes (300 bp, 400 bp, 500 bp and 600 bp) for *E. coli* DH1 and *S. Parasanguinis* FW213 are plotted against different depth of read coverage. A, B, C and D are N50 from Group A [PE] and E, F, G and H are N50 from Group A [SE] which are results from all reads assembled as paired-end reads and single end reads, respectively. N50 increases as the depth of read coverage increase and finally reach plateau. Different library sizes show difference in N50 values while the smallest library size (Lib_300) always gives the lowest N50 value. (TIFF 814 kb) Additional file 4: Figure S3. Number of misassemblies for different assembly strategies. Number of misassemblies for the *de novo* assembly results for *E. coli* DH1 and *S. Parasanguinis* FW213 are shown together with their standard errors of the mean. Group A [PE] and Group A [SE] represent all reads assembled as paired-end reads and single end reads, respectively. Group A [PE + SE] represents all the non-overlapped paired-end reads assembled together with merged reads. Group M [PE] and Group M [SE] represent Group M reads assembled as paired-end reads and single end reads, respectively. The numbers of misassemblies fluctuate a lot when depths of read number are low and gradually decreases until they reach a steady number. The paired-end reads (Group A [PE] and Group M [PE]) in S. *Parasanguinis* FW213 gave the lowest number of misassemblies when depths of read number are high. (TIFF 669 kb) Additional file 5: Figure S4. N50 for assembly results from different assembly strategies assembled by IDBA-UD and SPAdes. N50 values for the *de novo* assembly results for *E. coli* DH1 and *S. Parasanguinis* FW213 by IDBA-UD and SPAdes. Group A [PE] and Group A [SE] represent all reads assembled as paired-end reads and single end reads, respectively. Group A [PE + SE] represents all the non-overlapped paired-end reads assembled together with merged reads. Group M [PE] and Group M [SE] represent Group M reads assembled as paired-end reads and single end reads, respectively. Group M slightly outcompete Group A when depth of read coverage is low. However, Group A always has the highest N50 values when depth of read coverage is high. (TIFF 867 kb) Additional file 6: Figure S5. Percentage of genome covered by contigs assembled by different assembly strategies. Covered genome fraction are shown for library size 300 bp and 400 bp. Group A includes all reads without selection and Group M are read that containing overlapped region. The covered fraction increases as depth of read coverage increases. Higher percentage were found for Group M comparing to Group A when depth of read coverage are low. However, all strategies reach similar percentage of genome coverage when depths of read coverage are high. (TIFF 457 kb)
