# Supplementary material for: Is the whole greater than the sum of its parts? De novo assembly strategies for bacterial genomes based on paired-end sequencing
Source: BMC Genomics. 2015 Aug 28;16(1):648. doi: 10.1186/s12864-015-1859-8 (PMC4552406; doi:10.1186/s12864-015-1859-8)
Supplement: Additional file 1: Table S1-S7. — Table S1 – Summary of mapping reads to reference genome. Table S2 – Mismatch rates detected from reads mapped to reference genome before and after merge by different merge programs. Table S3 – Insertion rates detected from reads mapped to reference genome before and after merge by different merge programs. Table S4 – Deletions rates detected from reads mapped to reference genome before and after merge by different merge programs. Table S5 – Q30* values for Group A from different libraries. Table S6 – Q30* values for Group M from different libraries. Table S7 – Tandem repeats in S. Parasanguinis FW213. (DOCX 36 kb) [file 12864_2015_1859_MOESM1_ESM.docx]

# Additional files

### Table S1 – Summary of mapping reads to reference genome

|  |  | **Paired-end** |  |  | **trimmed reads*** | | | |  | | **raw reads** | | |  |
| --- | --- | --- | --- | --- | --- | --- | --- | --- | --- | --- | --- | --- | --- | --- |
| **Reference genome** |  | **library size** |  |  | **A [SE] ^**^** | **M [PE] ^**^** | **M [SE] ^**^** |  | | **A [SE] ^**^** | | **M [PE] ^**^** | **M [SE] ^**^** | |
| *E. coli* DH1 |  | 300 bp |  | mapping rate | 90.48% | 90.55% | 90.59% |  | | 89.93% | | 90.53% | 90.57% | |
|  |  | 400 bp |  |  | 90.45% | 90.43% | 90.48% |  | | 89.82% | | 90.59% | 90.64% | |
|  |  | 500 bp |  |  | 89.22% | 89.22% | 89.35% |  | | 88.57% | | 90.19% | 90.30% | |
|  |  | 600 bp |  |  | 89.97% | 81.80% | 82.20% |  | | 88.82% | | 82.09% | 82.28% | |
|  |  | 300 bp |  | mismatch rate | 1.80‰ | 1.69‰ | 1.38‰ |  | | 7.54‰ | | 5.48‰ | 1.80‰ | |
|  |  | 400 bp |  |  | 2.06‰ | 1.80‰ | 1.58‰ |  | | 8.64‰ | | 6.24‰ | 2.94‰ | |
|  |  | 500 bp |  |  | 1.99‰ | 1.69‰ | 1.65‰ |  | | 9.40‰ | | 5.38‰ | 4.83‰ | |
|  |  | 600 bp |  |  | 2.39‰ | 2.35‰ | 3.11‰ |  | | 11.45‰ | | 6.42‰ | 4.34‰ | |
| *S. parasanguinis* FW213 |  | 300 bp |  | mapping rate | 98.90% | 98.97% | 98.95% |  | | 98.29% | | 98.95% | 98.96% | |
|  |  | 400 bp |  |  | 96.53% | 97.43% | 97.43% |  | | 95.76% | | 97.65% | 97.66% | |
|  |  | 500 bp |  |  | 98.66% | 97.90% | 97.88% |  | | 97.96% | | 98.12% | 98.13% | |
|  |  | 600 bp |  |  | 98.25% | 97.70% | 97.66% |  | | 97.48% | | 97.90% | 97.83% | |
|  |  | 300 bp |  | mismatch rate | 1.81‰ | 1.74‰ | 1.41‰ |  | | 4.62‰ | | 3.61‰ | 1.71‰ | |
|  |  | 400 bp |  |  | 1.88‰ | 1.63‰ | 1.47‰ |  | | 4.90‰ | | 3.78‰ | 2.32‰ | |
|  |  | 500 bp |  |  | 2.05‰ | 1.71‰ | 1.81‰ |  | | 5.72‰ | | 3.65‰ | 3.23‰ | |
|  |  | 600 bp |  |  | 2.21‰ | 2.22‰ | 2.31‰ |  | | 6.64‰ | | 4.02‰ | 2.59‰ | |

* Reads were trimmed based on two criteria: quality Phred score larger than Q20 and length longer than 50 bp

** Group A includes all reads; Group M includes reads can be merged; SE: mapped as single-end reads; PE: mapped as paired-end reads

### Table S2 – Mismatch rates detected from reads mapped to reference genome before and after merge by different merge programs

|  |  | **Paired-end** |  | **trimmed reads*** | | | | |  | | **raw reads** | | | | |
| --- | --- | --- | --- | --- | --- | --- | --- | --- | --- | --- | --- | --- | --- | --- | --- |
| **Reference genome** |  | **library size** |  | **FLASH** | **PANDAseq** | **PEAR** | **Non-merged** |  | | **FLASH** | | **PANDAseq** | **PEAR** | **Non-merged** |  |
| *E. coli* DH1 |  | 300 bp |  | 1.38‰ | 1.64‰ | 1.40‰ | 1.80‰ |  | | 1.67‰ | | 2.02‰ | 2.70‰ | 7.54‰ |  |
|  |  | 400 bp |  | 1.58‰ | 2.64‰ | 1.62‰ | 2.06‰ |  | | 3.03‰ | | 4.99‰ | 5.26‰ | 8.64‰ |  |
|  |  | 500 bp |  | 1.75‰ | 4.92‰ | 2.33‰ | 1.99‰ |  | | 6.9‰ | | 11.29‰ | 7.79‰ | 9.40‰ |  |
|  |  | 600 bp |  | 3.36‰ | 3.62‰ | 4.85‰ | 2.39‰ |  | | 6.03‰ | | 7.84‰ | 7.71‰ | 11.45‰ |  |
| *S. parasanguinis* FW213 |  | 300 bp |  | 1.42‰ | 1.52‰ | 1.46‰ | 1.81‰ |  | | 1.67‰ | | 1.87‰ | 2.14‰ | 4.62‰ |  |
|  |  | 400 bp |  | 1.45‰ | 1.96‰ | 1.52‰ | 1.88‰ |  | | 2.56‰ | | 3.10‰ | 3.19‰ | 4.90‰ |  |
|  |  | 500 bp |  | 2.25‰ | 5.32‰ | 2.51‰ | 2.05‰ |  | | 4.21‰ | | 7.65‰ | 4.65‰ | 5.72‰ |  |
|  |  | 600 bp |  | 3.18‰ | 3.20‰ | 2.47‰ | 2.21‰ |  | | 4.00‰ | | 5.24‰ | 3.32‰ | 6.64‰ |  |
|  | | | | | | | | | | | | | | |  |

* Reads were trimmed based on two criteria: quality Phred score larger than Q20 and length longer than 50 bp

### Table S3 – Insertion rates detected from reads mapped to reference genome before and after merge by different merge programs

|  |  | **Paired-end** |  | **trimmed reads*** | | | | |  | | **raw reads** | | | | |
| --- | --- | --- | --- | --- | --- | --- | --- | --- | --- | --- | --- | --- | --- | --- | --- |
| **Reference genome** |  | **library size** |  | **FLASH** | **PANDAseq** | **PEAR** | **Non-merged** |  | | **FLASH** | | **PANDAseq** | **PEAR** | **Non-merged** |  |
| *E. coli* DH1 |  | 300 bp |  | 0.08‰ | 2.70‰ | 0.12‰ | 0.06‰ |  | | 0.06‰ | | 0.07‰ | 0.07‰ | 0.07‰ |  |
|  |  | 400 bp |  | 0.16‰ | 14.66‰ | 0.41‰ | 0.06‰ |  | | 0.07‰ | | 0.13‰ | 0.10‰ | 0.08‰ |  |
|  |  | 500 bp |  | 2.42‰ | 57.20‰ | 6.63‰ | 0.07‰ |  | | 0.31‰ | | 26.80‰ | 0.92‰ | 0.08‰ |  |
|  |  | 600 bp |  | 1.05‰ | 1.49‰ | 1.18‰ | 0.09‰ |  | | 4.17‰ | | 5.48‰ | 3.86‰ | 0.10‰ |  |
| *S. parasanguinis* FW213 |  | 300 bp |  | 0.09‰ | 0.68‰ | 0.10‰ | 0.07‰ |  | | 0.08‰ | | 0.09‰ | 0.08‰ | 0.08‰ |  |
|  |  | 400 bp |  | 0.23‰ | 5.76‰ | 0.23‰ | 0.08‰ |  | | 0.08‰ | | 0.15‰ | 0.12‰ | 0.09‰ |  |
|  |  | 500 bp |  | 3.46‰ | 61.14‰ | 7.06‰ | 0.08‰ |  | | 1.65‰ | | 39.42‰ | 2.57‰ | 0.09‰ |  |
|  |  | 600 bp |  | 1.58‰ | 3.72‰ | 1.08‰ | 0.08‰ |  | | 2.72‰ | | 8.89‰ | 2.01‰ | 0.09‰ |  |
|  | | | | | | | | | | | | | | |  |

* Reads were trimmed based on two criteria: quality Phred score larger than Q20 and length longer than 50 bp

### Table S4 – Deletions rates detected from reads mapped to reference genome before and after merge by different merge programs

|  |  | **Paired-end** |  | **trimmed reads*** | | | | |  | | **raw reads** | | | | |
| --- | --- | --- | --- | --- | --- | --- | --- | --- | --- | --- | --- | --- | --- | --- | --- |
| **Reference genome** |  | **library size** |  | **FLASH** | **PANDAseq** | **PEAR** | **Non-merged** |  | | **FLASH** | | **PANDAseq** | **PEAR** | **Non-merged** |  |
| *E. coli* DH1 |  | 300 bp |  | 0.01‰ | 0.01‰ | 0.01‰ | 0.01‰ |  | | 0.01‰ | | 0.01‰ | 0.01‰ | 0.03‰ |  |
|  |  | 400 bp |  | 0.01‰ | 0.02‰ | 0.01‰ | 0.01‰ |  | | 0.07‰ | | 0.06‰ | 0.05‰ | 0.03‰ |  |
|  |  | 500 bp |  | 0.02‰ | 0.04‰ | 0.04‰ | 0.01‰ |  | | 0.07‰ | | 0.21‰ | 0.10‰ | 0.03‰ |  |
|  |  | 600 bp |  | 0.03‰ | 0.02‰ | 0.07‰ | 0.01‰ |  | | 0.10‰ | | 0.04‰ | 0.16‰ | 0.03‰ |  |
| *S. parasanguinis* FW213 |  | 300 bp |  | 0.03‰ | 0.03‰ | 0.03‰ | 0.02‰ |  | | 0.03‰ | | 0.03‰ | 0.03‰ | 0.04‰ |  |
|  |  | 400 bp |  | 0.03‰ | 0.03‰ | 0.03‰ | 0.02‰ |  | | 0.08‰ | | 0.05‰ | 0.05‰ | 0.04‰ |  |
|  |  | 500 bp |  | 0.17‰ | 0.05‰ | 0.06‰ | 0.02‰ |  | | 0.06‰ | | 0.10‰ | 0.08‰ | 0.03‰ |  |
|  |  | 600 bp |  | 0.39‰ | 0.02‰ | 0.05‰ | 0.02‰ |  | | 0.47‰ | | 0.04‰ | 0.07‰ | 0.04‰ |  |
|  | | | | | | | | | | | | | | |  |

* Reads were trimmed based on two criteria: quality Phred score larger than Q20 and length longer than 50 bp

### Table S5 – Q30* values for Group A from different libraries

| **Reference genome** |  | **# of repeat** |  | **300 bp (%)** | **400 bp (%)** | **500 bp (%)** | **600 bp (%)** |
| --- | --- | --- | --- | --- | --- | --- | --- |
| ***E. coli* DH1** |  | 1 |  | 95.555 ± 0.008 | 95.191 ± 0.004 | 94.804 ± 0.007 | 94.108 ± 0.007 |
|  |  | 2 |  | 95.551 ± 0.013 | 95.191 ± 0.005 | 94.809 ± 0.013 | 94.093 ± 0.017 |
|  |  | 3 |  | 95.541 ± 0.011 | 95.187 ± 0.009 | 94.801 ± 0.010 | 94.104 ± 0.013 |
| ***S. parasanguinis* FW213** |  | 1 |  | 94.941 ± 0.520 | 96.142 ± 0.023 | 95.898 ± 0.018 | 95.258 ± 0.007 |
|  |  | 2 |  | 94.938 ± 0.524 | 96.143 ± 0.011 | 95.884 ± 0.015 | 95.268 ± 0.018 |
|  |  | 3 |  | 94.935 ± 0.517 | 96.131 ± 0.014 | 95.900 ± 0.017 | 95.250 ± 0.012 |

* The percentage of bases in the reads with a Phred score equal or larger than 30

### Table S6 – Q30* values for Group M from different libraries

| **Reference genome** |  | **# of repeat** |  | **300 bp (%)** | **400 bp (%)** | **500 bp (%)** | **600 bp (%)** |
| --- | --- | --- | --- | --- | --- | --- | --- |
| ***E. coli* DH1** |  | 1 |  | 95.846 ± 0.008 | 96.168 ± 0.009 | 97.086 ± 0.016 | 95.532 ± 0.116 |
|  |  | 2 |  | 95.848 ± 0.013 | 96.150 ± 0.013 | 97.076 ± 0.027 | 95.504 ± 0.175 |
|  |  | 3 |  | 95.836 ± 0.010 | 96.163 ± 0.011 | 97.093 ± 0.021 | 95.553 ± 0.149 |
| ***S. parasanguinis* FW213** |  | 1 |  | 96.283 ± 0.023 | 96.626 ± 0.015 | 97.075 ± 0.034 | 95.944 ± 0.125 |
|  |  | 2 |  | 96.284 ± 0.016 | 96.614 ± 0.020 | 97.102 ± 0.024 | 95.862 ± 0.060 |
|  |  | 3 |  | 96.269 ± 0.013 | 96.633 ± 0.018 | 97.085 ± 0.038 | 95.881 ± 0.042 |

* The percentage of bases in the reads with a Phred score equal or larger than 30

### Table S7 – Tandem repeats in *S. Parasanguinis* FW213

| **Position** |  | **Period size (bp)** |  | **Copy number** |
| --- | --- | --- | --- | --- |
| 1,975,924 –1,978,655 |  | 1,020 |  | 2.7 |
| 1,973,881 –1,976,571 |  | 981 |  | 2.7 |
| 99,885 – 102,190 |  | 711 |  | 3.2 |
| 1,596,299 –1,598,231 |  | 672 |  | 2.9 |
| 1,979,877 –1,981,322 |  | 672 |  | 2.2 |
| 1,988,757 –1,992,542 |  | 621 |  | 6.1 |
| 99,855 – 101,941 |  | 474 |  | 4.4 |
