# Supplementary figures and images for: Is the whole greater than the sum of its parts? De novo assembly strategies for bacterial genomes based on paired-end sequencing
Source: BMC Genomics. 2015 Aug 28;16(1):648. doi: 10.1186/s12864-015-1859-8 (PMC4552406; doi:10.1186/s12864-015-1859-8)

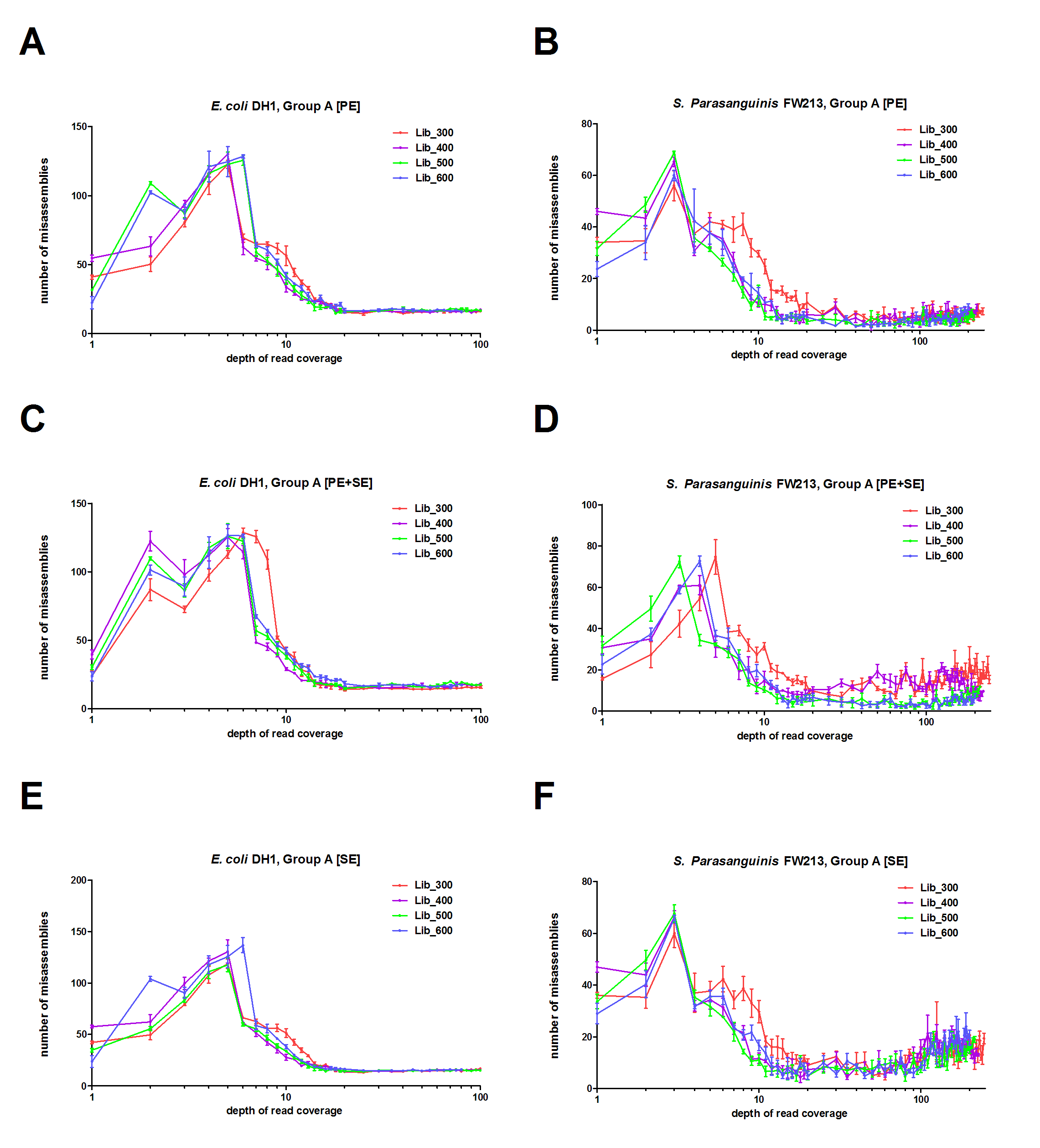

Supplement: Additional file 2: Figure S1. — Number of misassemblies for assembly results from different library sizes. Number of misassemblies for four different library sizes (300 bp, 400 bp, 500 bp and 600 bp) for E. coli DH1 and S. Parasanguinis FW213 are plotted against different depth of read coverage. A and B are from Group A [PE] which represents assembled results from all reads assembled as paired-end reads. C and D are from Group A [PE + SE] which represents assembled results from merged reads and all the non-overlapped paired end reads. E and F are from Group A [SE] which represents assembled results from all reads assembled as single-end reads. The number of misassemblies decreases as the depth of read coverage increases. Even though there are some fluctuations when depth of read coverage is low, the number of misassemblies reaches a steady number for all library sizes when depth of read coverage is high. (TIFF 882 kb) [file 12864_2015_1859_MOESM2_ESM.tif]

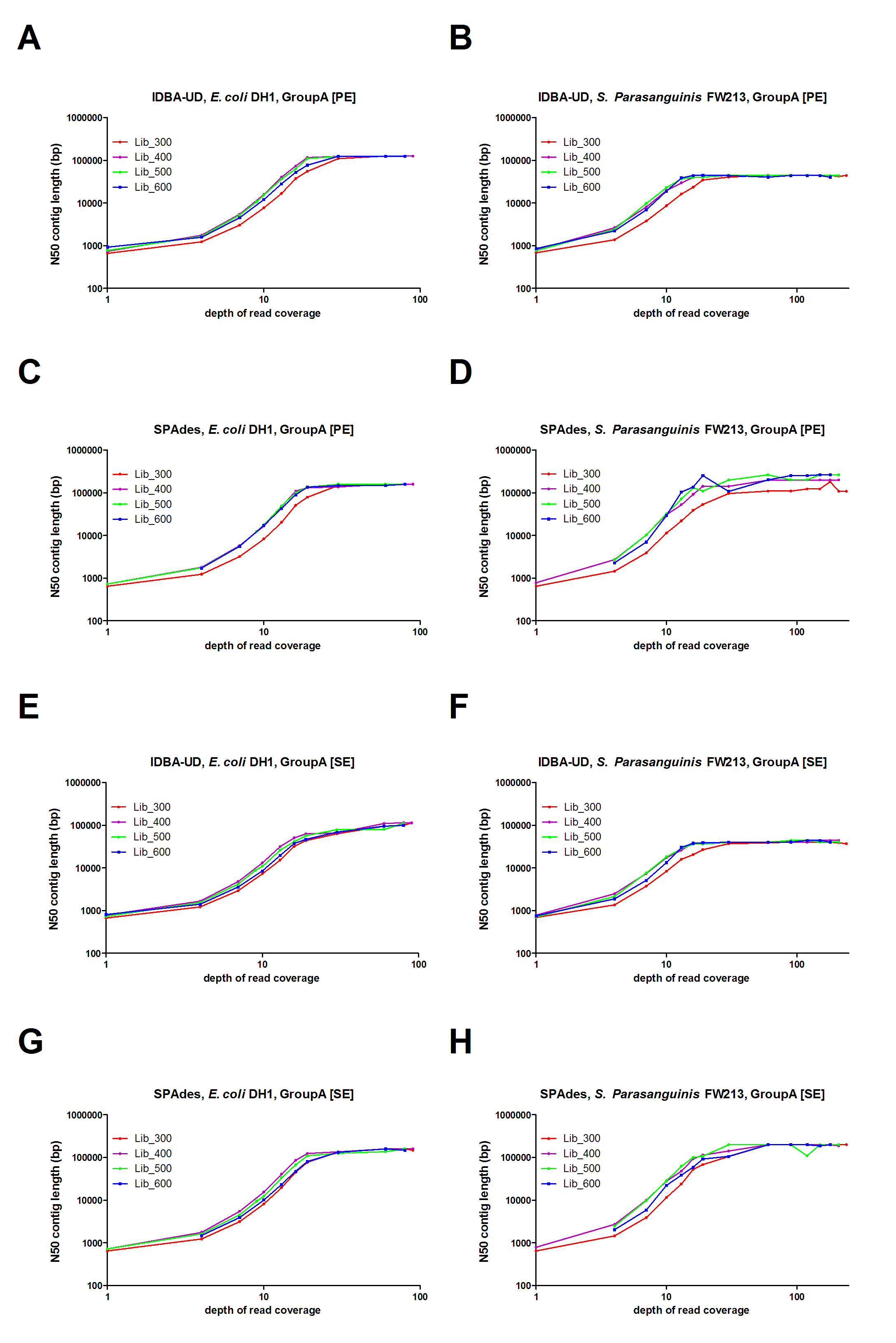

Supplement: Additional file 3: Figure S2. — N50 for assembly results from different library sizes assembled by IDBA-UD and SPAdes. N50 values for four different library sizes (300 bp, 400 bp, 500 bp and 600 bp) for E. coli DH1 and S. Parasanguinis FW213 are plotted against different depth of read coverage. A, B, C and D are N50 from Group A [PE] and E, F, G and H are N50 from Group A [SE] which are results from all reads assembled as paired-end reads and single end reads, respectively. N50 increases as the depth of read coverage increase and finally reach plateau. Different library sizes show difference in N50 values while the smallest library size (Lib_300) always gives the lowest N50 value. (TIFF 814 kb) [file 12864_2015_1859_MOESM3_ESM.tif]

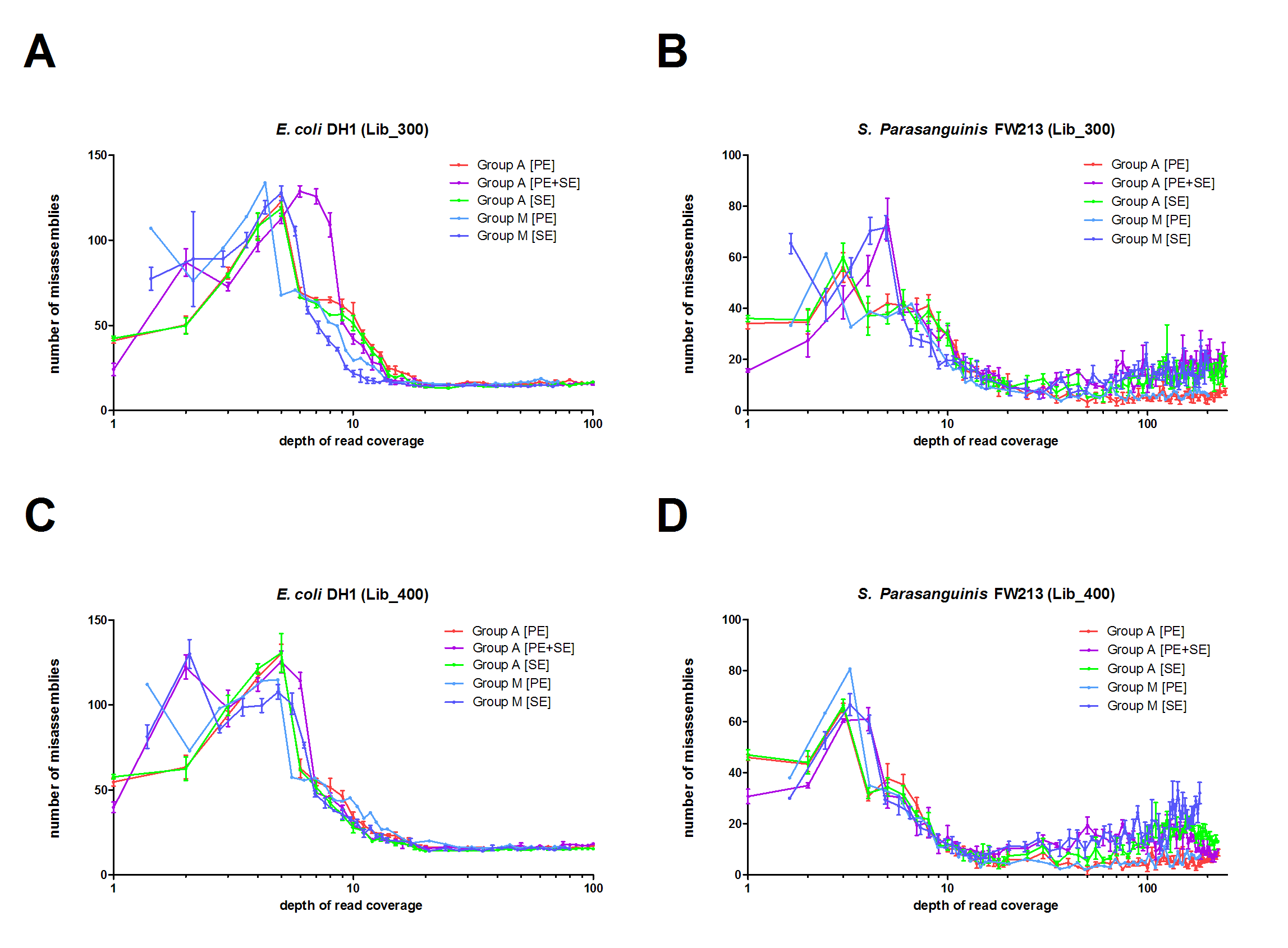

Supplement: Additional file 4: Figure S3. — Number of misassemblies for different assembly strategies. Number of misassemblies for the de novo assembly results for E. coli DH1 and S. Parasanguinis FW213 are shown together with their standard errors of the mean. Group A [PE] and Group A [SE] represent all reads assembled as paired-end reads and single end reads, respectively. Group A [PE + SE] represents all the non-overlapped paired-end reads assembled together with merged reads. Group M [PE] and Group M [SE] represent Group M reads assembled as paired-end reads and single end reads, respectively. The numbers of misassemblies fluctuate a lot when depths of read number are low and gradually decreases until they reach a steady number. The paired-end reads (Group A [PE] and Group M [PE]) in S. Parasanguinis FW213 gave the lowest number of misassemblies when depths of read number are high. (TIFF 669 kb) [file 12864_2015_1859_MOESM4_ESM.tif]

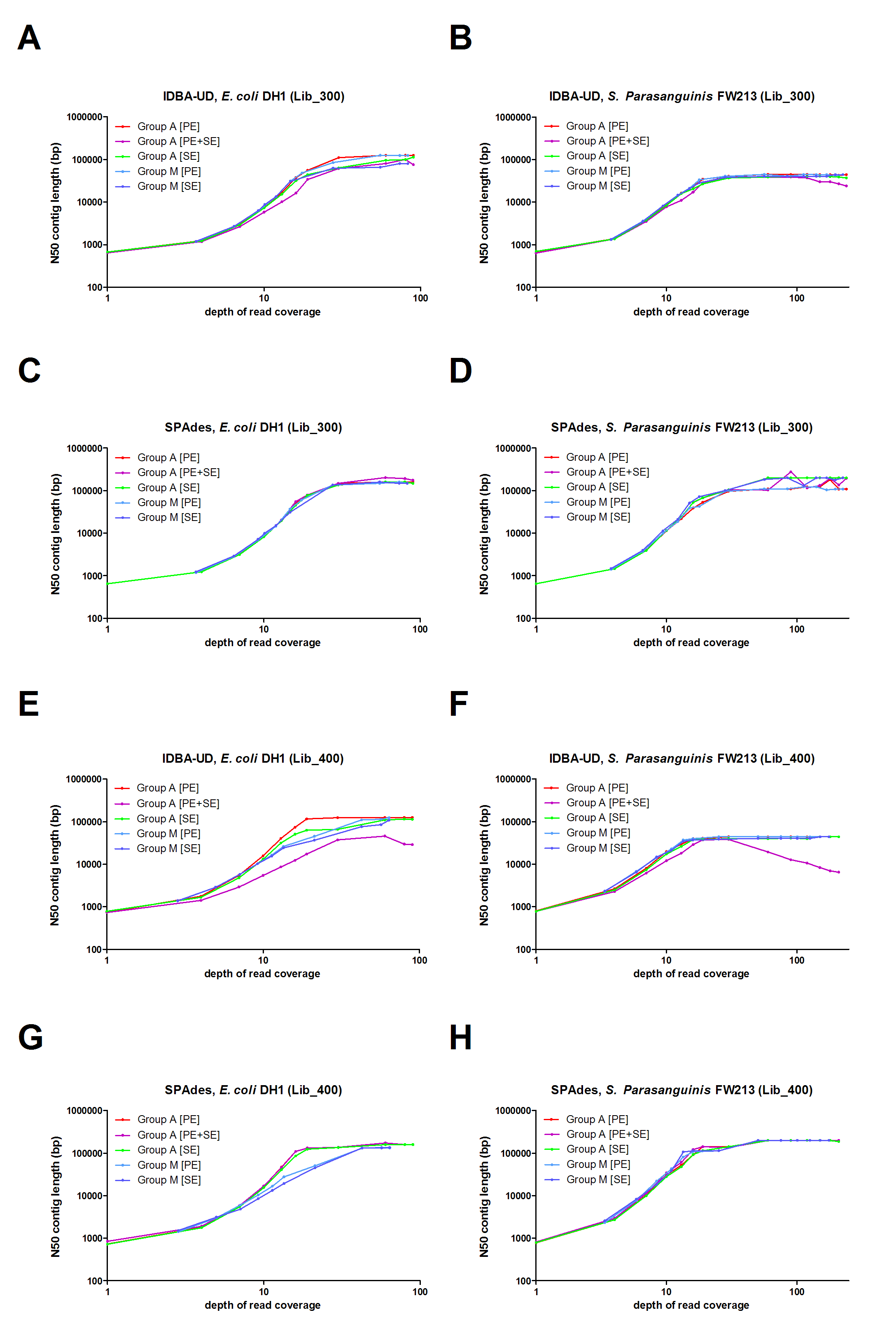

Supplement: Additional file 5: Figure S4. — N50 for assembly results from different assembly strategies assembled by IDBA-UD and SPAdes. N50 values for the de novo assembly results for E. coli DH1 and S. Parasanguinis FW213 by IDBA-UD and SPAdes. Group A [PE] and Group A [SE] represent all reads assembled as paired-end reads and single end reads, respectively. Group A [PE + SE] represents all the non-overlapped paired-end reads assembled together with merged reads. Group M [PE] and Group M [SE] represent Group M reads assembled as paired-end reads and single end reads, respectively. Group M slightly outcompete Group A when depth of read coverage is low. However, Group A always has the highest N50 values when depth of read coverage is high. (TIFF 867 kb) [file 12864_2015_1859_MOESM5_ESM.tif]

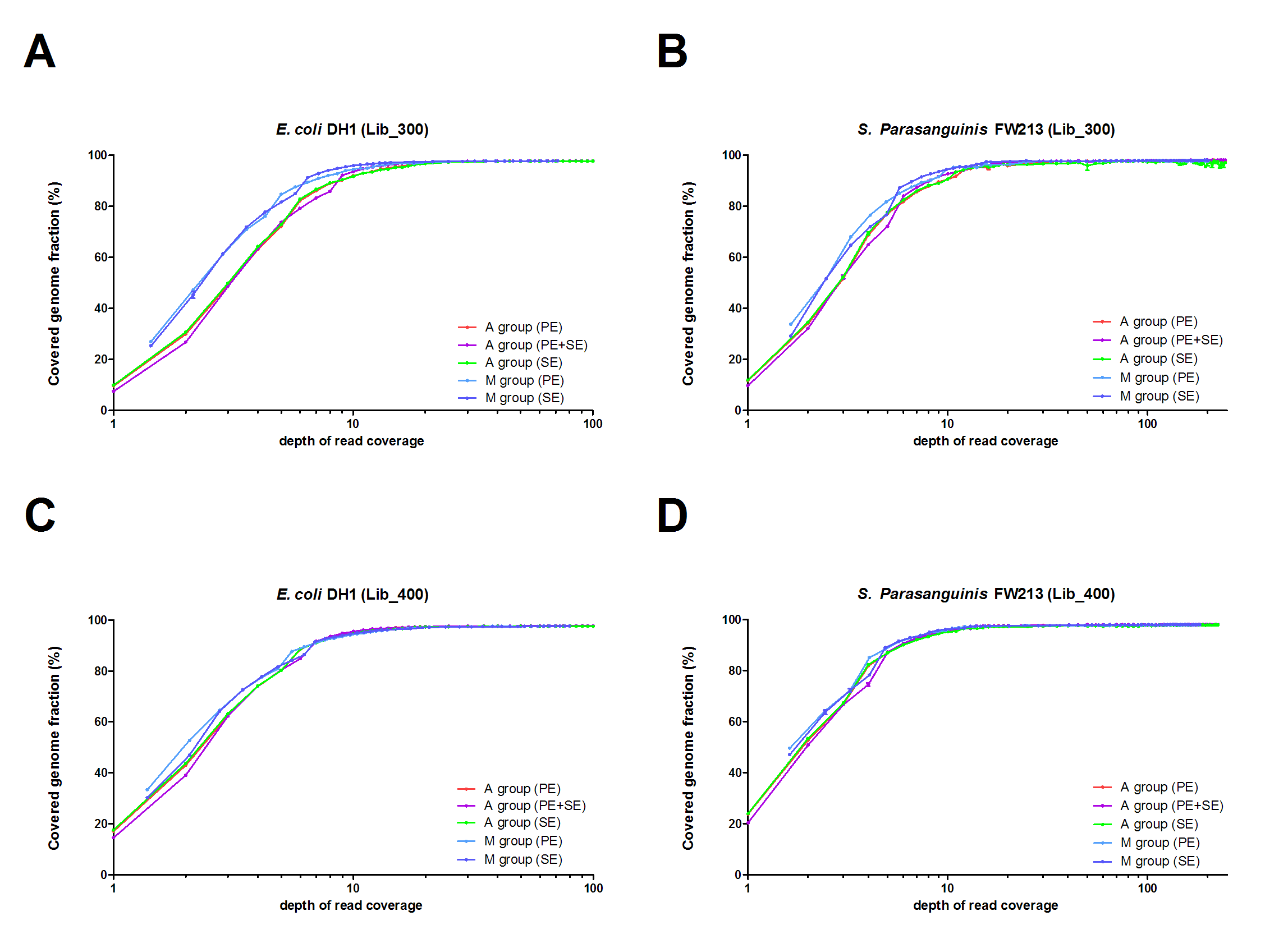

Supplement: Additional file 6: Figure S5. — Percentage of genome covered by contigs assembled by different assembly strategies. Covered genome fraction are shown for library size 300 bp and 400 bp. Group A includes all reads without selection and Group M are read that containing overlapped region. The covered fraction increases as depth of read coverage increases. Higher percentage were found for Group M comparing to Group A when depth of read coverage are low. However, all strategies reach similar percentage of genome coverage when depths of read coverage are high. (TIFF 457 kb) [file 12864_2015_1859_MOESM6_ESM.tif]
